# β-Phase Crystallinity, Printability, and Piezoelectric Characteristics of Polyvinylidene Fluoride (PVDF)/Poly(methyl methacrylate) (PMMA)/Cyclopentyl-Polyhedral Oligomeric Silsesquioxane (Cp-POSS) Melt-Compounded Blends

**DOI:** 10.1021/acsapm.4c00468

**Published:** 2024-05-14

**Authors:** Toby R. Edwards, Rahul Shankar, Paul G. H. Smith, Jacob A. Cross, Zoe A. B. Lequeux, Lisa K. Kemp, Zhe Qiang, Scott T. Iacano, Sarah E. Morgan

**Affiliations:** † School of Polymer Science and Engineering, University of Southern Mississippi, 118 College Drive, #5050, Hattiesburg, Mississippi 39406, United States; ‡ Department of Chemistry and Chemistry Research Center, United States Air Force Academy, 2355 Fairchild Drive, Suite 2N225, Colorado Springs, Colorado 80840, United States

**Keywords:** poly(vinylidene fluoride), PVDF, layer-based material extrusion (MEX), 3D printing, piezoelectric, polyhedral oligomeric silsesquioxane (POSS)

## Abstract

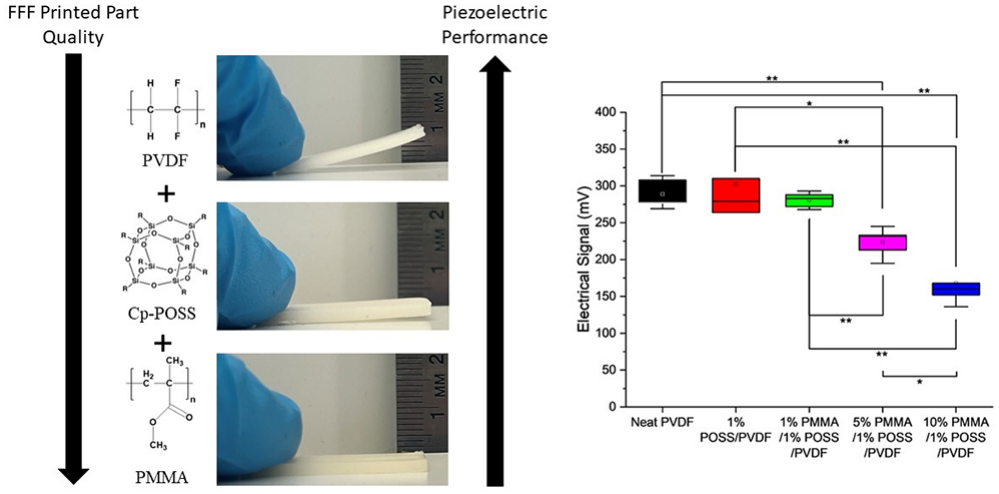

Poly(vinylidene fluoride) (PVDF) is a semicrystalline polymer that exhibits unique piezoelectric characteristics along with good chemical resistance and high thermal stability. Layer-based material extrusion (MEX) 3D printing of PVDF is desired to create complex structures with piezoelectric properties; however, the melt processing of PVDF typically directs the formation of the α crystalline allomorph, which does not contribute to the piezoelectric response. In this work, PVDF was compounded with poly(methyl methacrylate) (PMMA) and cyclopentyl-polyhedral oligomeric silsesquioxane (Cp-POSS) nanostructured additives in binary and ternary blends to improve MEX printability while maintaining piezoelectric performance. Overall crystallinity and β phase content were evaluated and quantified using a combination of attenuated total reflectance-Fourier transform infrared spectroscopy (ATR-FTIR) and differential scanning calorimetry (DSC). Enhancement of MEX printability was measured by quantifying the interlayer adhesion and warpage of printed parts. All blends studied contained a significant percentage of β allomorph, but it could be detected by ATR-FTIR only after the removal of a thin surface layer. Inclusion of 1% Cp-POSS and up to 10% PMMA in blends with PVDF improved interlayer adhesion (2.3–3.6x) and lowered warpage of MEX printed parts compared to neat PVDF. The blend of 1% Cp-POSS/1% PMMA/PVDF was demonstrated to significantly improve the quality of MEX printed parts while showing similar piezoelectric performance to that of neat PVDF (average piezoelectric coefficient 24 pC/N). MEX printing of PVDF blends directly into usable parts with significant piezoelectric performance while reducing the challenges of printing the semicrystalline polymer opens the potential for application in a number of high value sectors.

## 1 Introduction

Polyvinylidene fluoride (PVDF) is a specialty thermoplastic known for its remarkable piezoelectric and pyroelectric properties, making it attractive for smart sensing, actuation, and energy harvesting applications.^1−3^ As a semicrystalline thermoplastic, the broad temperature range (∼200 °C) between its glass transition and melting temperatures allows PVDF to be processed using traditional melt processing operations, such as extrusion and injection molding, along with additive manufacturing techniques like layer-based material extrusion (MEX).^3^ However, its high melting temperature and slow crystallization characteristics pose challenges to its widespread applicability for high throughput processes.^4−6^ Moreover, the high degree of thermal expansion and contraction of semicrystalline feed materials during MEX printing results in poor layer-to-layer adhesion quality and a high degree of warpage, making it difficult for PVDF to compete with conventional MEX feedstock materials like polylactic acid (PLA) and acrylonitrile butadiene styrene (ABS).^3,7^ Precise control of the MEX printability and crystallization properties of PVDF are key for achieving maximum performance and expanding applications.

PVDF exhibits a unique crystalline polymorphism characterized by three major phases which differ in the conformation of the backbone: α (trans–gauche; TGTG’), β (trans–trans; TTTT) and γ (T_3_GT_3_G). Of these, the β-phase, which is the thermodynamically metastable state, displays the highest net dipole moment and is responsible for the piezoelectric characteristics of PVDF.^8−10^ However, the nonpolar α-phase is thermodynamically most favorable, particularly during crystallization from the melt state, which has hindered MEX printing applications.^11−13^ Utilization of the piezoelectric nature of PVDF along with 3D printing enables expansion of the material to a wide range of applications such as sensors, actuators, and energy harvesting.^14,15^ Research efforts have focused on conversion of the α allomorph to the β allomorph through the use of poling, cold drawing, and thermal annealing.^16−19^ For example, Zhou et al. demonstrated the use of biaxial stretching to increase β phase and crystallinity in polymer films prepared by extrusion and casting.^20^ Tao and co-workers prepared PVDF films by MEX printing and reported increased β phase and piezoelectric charge coefficient after postprinting stretching and poling processes.^21^ These reports demonstrate the ability to manipulate the crystallinity of melt-processed PVDF by postprocessing methods, but they add process complexity and may have limited scalability. Zhang et al. provided a review of recent advancement in PVDF piezoelectricity and the impacts of crystallinity and highlighted current limitations to creating flexible and complex structures.^22^

Alternatives such as blending of PVDF with poly(methyl methacrylate) (PMMA) have also been explored. In solution blends, PMMA has been shown to increase PVDF β crystalline phase content.^23−26^ In the melt, PMMA is reported to be miscible with PVDF at all composition levels as a result of hydrogen bonding between the carbonyl group of PMMA and the acidic hydrogens in the PVDF backbone, and dipole–dipole associations between the CH_2_ group of PMMA and CF_2_ group of PVDF.^27−29^ Leonard and co-workers studied the impact of PMMA tacticity and loading level in melt blends with PVDF and reported that the β-phase content was increased with increasing PMMA ratio after an isothermal crystallization step.^30^ Aid et al. studied PVDF/PMMA melt blends and reported an increase in the percentage of the β allomorph with the inclusion of 10 wt % PMMA, but the β-phase content dropped at 30 wt % PMMA.^27^ Friere et al. reported that increasing PMMA content improved the processability of PVDF and that high shear mixing promotes a greater degree of interaction between the polymers and assists in the ability of the molecular segments to diffuse to the crystal growth front.^31^ While these limited reports show promise for PVDF/PMMA blends, reported results are not in full agreement, most likely due to differences in processing and characterization methods.

Another strategy to influence PVDF crystallization and processability is the incorporation of low concentrations of polyhedral oligomeric silsesquioxane (POSS) nanostructured additives. The hybrid structure of POSS enables enhanced thermal stability, mechanical robustness, wear resistance, and oxidative stability through the robust inorganic cage, while the tunable organic corona promotes favorable interactions and compatibility with a polymer matrix. At low loadings, POSS has been shown to provide a lubricating effect in multiple polymer blends, by our group and others.^32−34^ In many semicrystalline polymers, POSS has also been shown to act as a nucleating agent which aids in increasing the overall degree of crystallinity as well as the rate of crystallization.^35−37^ Most reports to date of PVDF/POSS have involved fluoroalkyl-substituted POSS solution blends with PVDF, in which improvements in mechanical, crystalline, and dielectric properties were observed.^38−41^ Limited reports of PVDF/nonfluorinated POSS melt blends have appeared. Martins and co-workers studied blends prepared in a batch melt mixer of PVDF with up to 5 wt % methacrylate-substituted POSS and reported viscosity reductions at all loading levels.^42^ At POSS concentrations above 1 wt %, nanoparticle aggregation and increases in both α and β allomorphs were observed. It should be noted that in this study by Martins, β fraction is calculated from a small shoulder on the α peak, rather than as the separate peak generally reported by others.^43^ In a separate work, Martins et al. reported a reduction in the crystallization rate and a small increase in the interlamellar amorphous phase in PVDF blends with 5 wt % POSS.^44^ Recently, Joshi et al. reported that the addition of zinc oxide nanofiller increased the percent β-phase in MEX printed PVDF.^45^ These studies indicate the potential of PVDF/POSS blends for use in high throughput additive manufacturing processes like MEX printing. However, there is a need for further understanding of the relationships between rheology, processing, crystallization, and properties.

The focus of this study is to determine the effects of PMMA and POSS incorporation on the rheology and crystallization behavior of PVDF melt blends with the goals of improving processability while maintaining β-phase content for MEX printing and piezoelectric applications without the need for additional postprinting process operations. Cyclopentyl-POSS (Cp-POSS) and PMMA homopolymer were compounded with PVDF in a twin-screw extruder to form a series of binary and ternary blends. Differential scanning calorimetry (DSC) was used to determine the overall crystalline content. Attenuated total reflectance Fourier-transform infrared spectroscopy (ATR-FTIR) was used to evaluate α- and β-phase content of films prepared via compression molding. Melt processability was evaluated through dynamic rheological experiments which examined the effect of shear rate on the melt flow properties. The performance and applicability of the blends were evaluated by characterizing sample warpage, interlayer adhesion, and the piezoelectric effect of MEX printed parts. The efficacy of PMMA and Cp-POSS as drop-in additives for melt blending with PVDF to achieve improved printability while maintaining piezoelectric performance was determined and will enable their utilization in advanced manufacturing processes.

## 2 Experimental Section

### 2.1 Materials

Poly(vinylidene fluoride) (PVDF pellets, Kynar 705), a high flow formulation, was donated by Arkema Chemical in pellet form with a reported density of 1.78 g cm^–1^ and a melt volume-flow rate of 40 cm^3^/10 min at 230 °C. Cyclopentyl polyhedral oligomeric silsesquioxane (Cp-POSS) was purchased from Hybrid Plastics (Hattiesburg, MS) as a white powder and used as received (Figure S1). Poly(methyl methacrylate) (PMMA, molecular weight: 120 kDa) was purchased from Sigma-Aldrich.

### 2.2 Methods

#### Compounding and Sample Preparation

PVDF was compounded with PMMA and Cp-POSS using a Thermo Fisher Process 11 corotating twin screw extruder with an 11 mm screw diameter and 40 L/D. Compounding was conducted at 100 rpm with barrel temperature increasing from 190 °C at feed to 230 °C at the extrusion dye. Sample blends were premixed in a sealed container using a roll mill prior to compounding. Polymer blends were fed into the extruder using a single screw volumetric feeder operated at 12 rpm. The extruded strands were cooled using a Thermo Fisher mini air-cooled conveyor belt with compressed air blowing across the belt and filament diameter was controlled for each blend by adjusting the speed of the conveyor to obtain constant diameter of 2.5 ± 0.36 mm. Both MEX filament and pellets were made using this method, and pellets were cut to a length of ∼2 mm using the Thermo Fisher pelletizer. All compression molding was conducted using a Carver heated lab press with 152 × 152 mm steel plates. Molded samples were made from pellets of each compounded PVDF blend. Pellets were added to a 25 mm stainless steel circular mold with 1 mm of thickness and molded at a temperature of 220 °C with either 80 psi of pressure or no added pressure. After molding, samples were placed either into an ice bath or allowed to cool slowly in the melt press. Compounding parameters are provided in Table S1.

#### 3D Printing

Specimens were printed using a Lulzbot Taz Mini 2 layer-based material extrusion (MEX) 3D printer with a Lulzbot HE 0.5 mm hardened steel nozzle at a nozzle temperature of 230 °C, bed temperature of 80 °C, layer height of 0.2 mm, print speed of 20 mm/s, and a fan speed of 10%. Filament diameter was held constant at 2.5 ± 0.36 mm. To ensure adhesion to the print bed, Vision Miner Nano Polymer adhesive was applied prior to printing, and all specimens were printed with a 1 cm brim. Specimens measuring 3 mm by 12 mm by 70 mm were printed for piezoelectric testing, with 2 walls, 4 top and bottom layers, and a “grid” infill pattern with alternating raster angles of 45° and 135° at an infill percentage of 80%. Vertically printed rectangular samples for interlayer adhesion testing were printed using the same parameters as piezoelectric specimens with dimensions of 40 mm (length) by 10 mm (width) by 3 mm (thickness). A schematic of the sample preparation process is provided in Figure S2. A table of all printing parameters is provided in Table S2.

#### Fourier Transform Infrared Spectroscopy

FT-IR analysis was performed using a Thermo Nicolet 8700 spectrophotometer equipped with a Smart iTR Attenuated Total Reflectance (ATR) accessory. Samples collected in transmittance mode used a KBr beam splitter and DTGS TEC detector under a nitrogen atmosphere. Data was collected at a resolution of 2 cm^–1^ with 64 scans. Samples were analyzed from 2000 to 600 cm^–1^ to determine the relative fractions of α, β, and γ crystalline phases. Spectra were baseline corrected using the built-in baseline correction tool in Thermo Omnic software. F_β-phase_ is the fraction of the β-phase polymorph present within the crystalline domains of PVDF and was calculated via the FTIR procedure outlined by Cai et al., where the intensity of the electroactive peak (*I*_*EA*_) at 840 cm^–1^ represents the sum of contributions from the β and γ phases, and the peak at 763 cm^–1^ represents contributions solely from the α phase.^43^ The ratio of β and γ phases was determined via comparison of peak intensities at 1275 and 1234 cm^–1^. Gamma phase concentration was negligible and is not reported in the Results and Discussion.

#### Depth Profile

A PVDF sample was prepared via compression molding into a 25 mm diameter disk and slowly cooled in the melt press. The disk was separated into two halves: one that was sanded and one that was taped to be used as the control. Half of the disk was sanded by moving the sample across 150 mm of 300 grit sandpaper 5 times. Ten levels of sanding, each consisting of a set of 5 passes across the sandpaper, were prepared, and evaluated by FTIR-ATR to determine F_β-phase_ as a function of depth. The depth of each level was determined by measuring the difference between the unsanded and sanded halves of the sample using a profilometer.

#### Differential Scanning Calorimetry (DSC)

The melting and crystallization properties of neat PVDF and the blends were determined with a Discover 250 DSC (TA Instruments) equipped with a Minichiller 300 (Huber, Offenburg, Germany) under nitrogen (flow rate = 50 mL min^–1^). Samples were heated to 220 °C, and the melting (*ΔH*_*f*_) endotherm was used to calculate the percent crystallinity of the samples, which was normalized by the weight fraction of PVDF within the blends.

#### Dynamic Mechanical Analysis (DMA)

DMA characterization was carried out on a Discovery 850 Dynamic Mechanical Analyzer (TA Instruments, New Castle, DE) using the film-tension mode. Rectangular specimens 35 mm by 12.8 mm were cut from 0.6 mm thick compression molded films. Temperature ramp tests were conducted by applying a sinusoidal load at a frequency of 1 Hz, which was superimposed on a static load maintained at 125% of the dynamic load, thus ensuring that the loading probe always remained in contact with the specimen. The tests were conducted at a constant strain of 0.01%. Each sample was scanned from 30 to 200 °C at a rate of 2 °C min^–1^ under air.

#### Small Amplitude Oscillatory Shear Rheology (SAOS)

Rheological evaluations were conducted on melt-pressed circular discs of 25 mm diameter and 2 mm thickness using a strain-controlled ARES rheometer (TA Instruments, New Castle, DE) equipped with an environment controller. Measurements were performed using a 25 mm parallel plate geometry at a gap of 1 mm. Isothermal frequency sweep tests (0.1–100 rad s^–1^, 5 points per decade) were conducted within the linear viscoelastic region (LVR) at a strain level of 1% with a temperature of 220 °C. Prior to the measurements, the rheometer was heated to the required temperature and allowed to equilibrate at the set gap. The samples were then loaded between the plates and held for approximately 20 min before starting the measurements.

#### Atomic Force Microscopy (AFM)

AFM samples were prepared by cutting extruded strands, encasing them in Gorilla epoxy to form bullets, and drying them overnight in ambient conditions. The bullets were microtomed using a glass knife at −30 °C with a speed of 1.5 mm/s and a feed of 200 nm in a Leica EM UC7 cryotome chamber attached to a Leica EM FC6 microtome until the surface of the polymer appeared glassy. A Dimension Icon AFM (Bruker) with NanoScope 8.15r3sr9 software was used to collect AFM images with a sharp silicon nitride cantilever (RTESP-300, nominal tip radius 8 nm; nominal resonance frequency of 300 kHz; nominal spring constant of 40 N/m) and a standard probe holder under ambient conditions with 512 × 512 data point resolution. AFM height and phase images were obtained simultaneously using standard tapping mode, and images were analyzed using NanoScope Analysis 1.50 software.

#### Scanning Electron Microscopy with Energy Dispersive X-ray Spectroscopy (SEM-EDX)

Samples were analyzed using a Zeiss Ultra 60 field emission scanning electron microscope with an accelerating voltage of 17 kV under vacuum. Standard surface images along with elemental mapping were obtained for each sample.

#### Wide Angle X-ray Scattering (WAXS)

A Xeuss 2.0 laboratory beamline (Xenocs Inc.) with an X-ray wavelength of 0.154 nm and sample-to-detector distances of 2.5 m was used to perform WAXS. Samples were fabricated using compression molding to form samples with ∼1 mm thickness and data was processed with IgorPro software.

#### Interlayer Adhesion

MEX vertically printed adhesion samples were evaluated using a Mark-10 tensile tester with a 250N load cell. Following ASTM D638, wedge grips (25 mm) were used to attach samples to the test frame, and an elongation rate of 20 mm/min was used for all experiments. Samples were elongated with force applied perpendicular to the printed layers, and the maximum force needed to separate the layers was measured. Five replicates of each blend were tested.

#### Piezoelectric Testing

The surface of the 3D printed samples was removed via sanding prior to testing. Samples were painted with silver paint to create a conductive network, and copper tape electrodes were applied to opposite sides of each sample. The copper electrodes were clamped with alligator clips and connected to a Rigol DS1104 Z-plus oscilloscope. Samples were mechanically deformed using a BYK Gardner impact tester with a 1.8 kg weight dropped from a height of 40 mm. The electrical response was recorded using the oscilloscope, and the maximum voltage output for each of the five replicates per MEX printed part was recorded. A diagram of the sample preparation and testing setup is provided in Figure S3.

## 3 Results and Discussion

### 3.1 β Allomorph Fraction, Percent Crystallinity, and Total β-Phase Content in PVDF Blends

**Figure 1:**
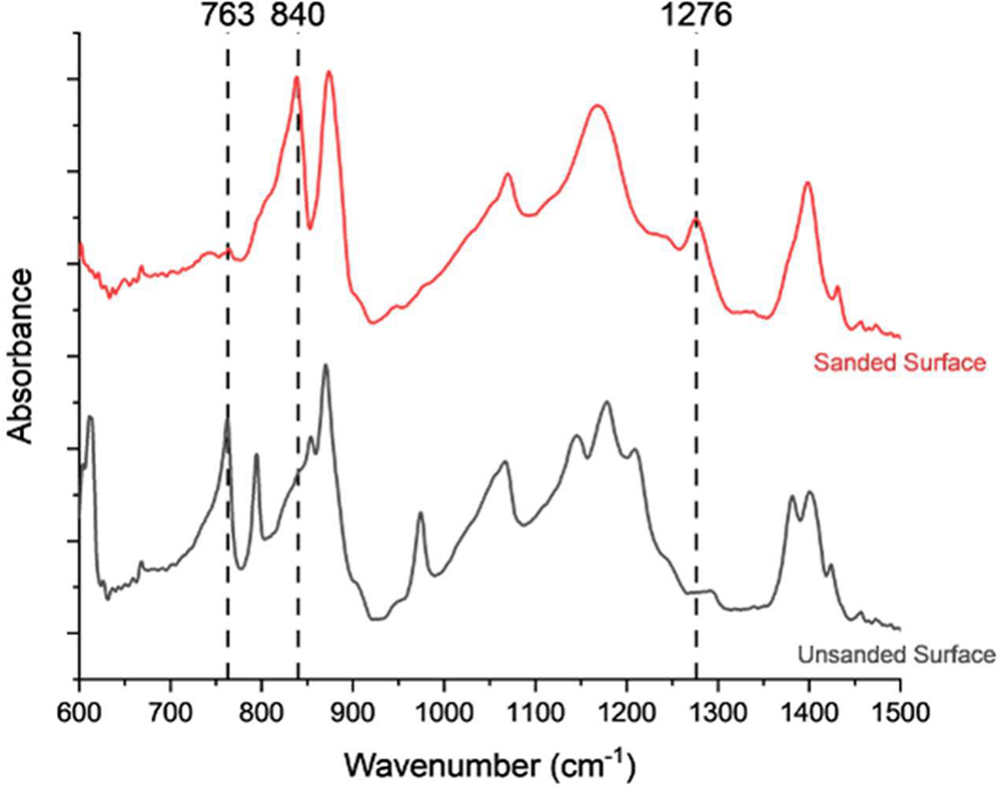
ATR-FTIR of compression molded PVDF before and after sanding to remove the surface layer. The sanded materials show characteristic β-phase peaks at 840 and 1276 cm^–1^, not present on the surface of the as-molded material.

Initially, ten blends of PVDF were compounded with 1–20 wt % PMMA and/or 1–5 wt % Cp-POSS as described in the Experimental Section. To explore potential differences in bulk and surface properties, samples were sanded to remove the surface layer, and both as-molded and sanded samples were characterized. Percentages of each crystalline PVDF allomorph can be determined using characteristic absorption peaks within the infrared region. ATR-FTIR spectra of compression molded neat PVDF samples before and after sanding to remove the surface layer are shown in Figure 1. As is expected for melt processed PVDF, the predominant polymorph present on the surface of the as-molded sample is α-phase, represented by a characteristic absorbance peak at 763 cm^–1^. Interestingly, after the surface layer is removed, absorbances consistent with the β allomorph are observed at 840 and 1276 cm^–1^. Similar results were obtained for each of the PVDF blends with PMMA and Cp-POSS (Figure S4). ATR-FTIR evaluation of the cross-section of a cryofractured extruded strand showed a similar percentage of β allomorph (63%), indicating that β-phase forms in the bulk and is not mechanically induced by the sanding process. The thickness of the surface layer (∼15 μm) was determined by removing layers and tracking changes in F_β_ (Figure S5).

The fraction of the β polymorph within the crystalline phase, F_β-phase_, can be determined using absorption intensities of characteristic peaks for β(I_840_) and α(I_763_) allomorphs (eq 1).^43^ The molar absorption coefficients, K_840_ = 7.7 × 104 cm^2^ mol^–1^ and K_763_ = 6.1 × 104 cm^2^ mol^–1^, were obtained from literature.^43^
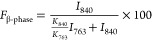
1

Profilometry indicated that the all-α surface layer is <15 μm thick, and removing the surface layer revealed that the β-phase allomorph is present in all samples at fractions greater than 50% (Table 1).

**Table 1:** PVDF Crystallinity, F_β-phase_, and Total β-Phase Content in Extruded and Air Cooled Pellets: (a) Neat PVDF and PMMA/PVDF Binary Blends and (b) Binary and Ternary Blends Containing PMMA and Cp-POSS

(a)				(b)			
Sample	Normalized PVDF *X*_c_	F_β-phase_	Total β-phase content	Sample	Normalized PVDF *X*_c_	F_β-phase_	Total β-phase content
Neat PVDF	55%	68%	37%	1% POSS/PVDF	57%	65%	36%
1% PMMA/PVDF	54%	62%	33%	5% POSS/PVDF	54%	65%	33%
5% PMMA/PVDF	53%	65%	33%	1% POSS/10% PMMA/PVDF	57%	53%	27%
10 wt % PMMA/PVDF	55%	61%	30%	5% POSS/10% PMMA/PVDF	59%	51%	25%
20 wt % PMMA/PVDF	54%	54%	24%	5% POSS/20% PMMA/PVDF	57%	49%	21%

Percent crystallinity (*X*_c_) normalized for PVDF content was determined by eq 2:
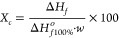
2where Δ*H*_f_ is the melt enthalpy of the blend determined by DSC (Figure S6), Δ*H*_0f100%_ is the melt enthalpy of the pure PVDF, and w is weight fraction of PVDF. Total β-phase content represents the percent of β polymorph in the entire blend (both crystalline and amorphous).

Results for neat PVDF and PMMA binary blends prepared by extrusion and air cooling are shown in Table 1a. The addition of PMMA does not impact the normalized PVDF crystallinity but does reduce the F_β-phase_ and the total β-phase content, with more pronounced effects as PMMA concentration increases. The decrease in total β-phase content is anticipated because the PMMA increases the amorphous content of the blends.

Table 1b includes binary and ternary blends containing Cp-POSS nanostructured additives. Introduction of Cp-POSS shows very little impact on crystallinity, F_β-phase_, and total β-phase content, particularly at 1 wt % loading. Ternary blends with PMMA and Cp-POSS display trends similar to those observed for the PMMA binary blends, with increasing loading levels of amorphous PMMA decreasing the total β-phase content.

To investigate the effect of cooling rate on degree of crystallinity and β allomorph content, samples were prepared by 1) cooling slowly in the melt press and 2) quenching in an ice water bath. The degree of crystallinity, F_β-phase_, and total β-phase content after crystallization under the two conditions are shown for neat PVDF, a binary blend with 1 wt % Cp-POSS, and a ternary blend with 10 wt % PMMA/1 wt % Cp-POSS in Table S3. Slow cooled samples show higher percent crystallinity than quenched samples, as is generally expected, but the F_β-phase_ is independent of cooling rate.^46^ Because the overall crystallinity is higher, the total β-phase content of the slow cooled samples is also higher.

Previous reports indicated that only the α allomorph forms during PVDF crystallization from the melt, and post processing, such as such as polling, cold drawing, and/or higher pressure annealing, is needed to form the β allomorph.^11,16,47,48^ Our studies show the β allomorph to be present in all samples, when evaluated via ATR-FTIR after the removal of the all-α surface layer, regardless of PMMA and/or Cp-POSS addition or crystallization condition. To our knowledge, this is the first report of a difference between the polymorphism of the surface layer and the bulk of melt processed PVDF. The most common technique used to differentiate allomorphs of PVDF is ATR-FTIR, which penetrates to depths smaller than the 15 μm surface layer,^49^ and thus only α allomorph was observed for the as-molded surface. In our study, for samples where the surface layer was removed, the ATR-FTIR spectra are similar to other published results of PVDF films made from solution casting or postprocessed melt blends, with a prominent β-phase peak (not a shoulder) at 840 cm^–1^.^18^ Our demonstration that the β allomorph is always present in the bulk provides a new perspective on previous reports focusing on the melt processing of PVDF. Analyzing the F_β-phase_ after the removal of the surface layer of melt compounded PVDF blends, our results indicate that PMMA addition does not increase the β-phase crystallinity. The presence of β-phase after crystallization from the melt in all samples allows for the fabrication of piezoelectric materials using common melt processing techniques such as MEX printing.

### 3.2 Viscosity and Shear Thinning

**Figure 2:**
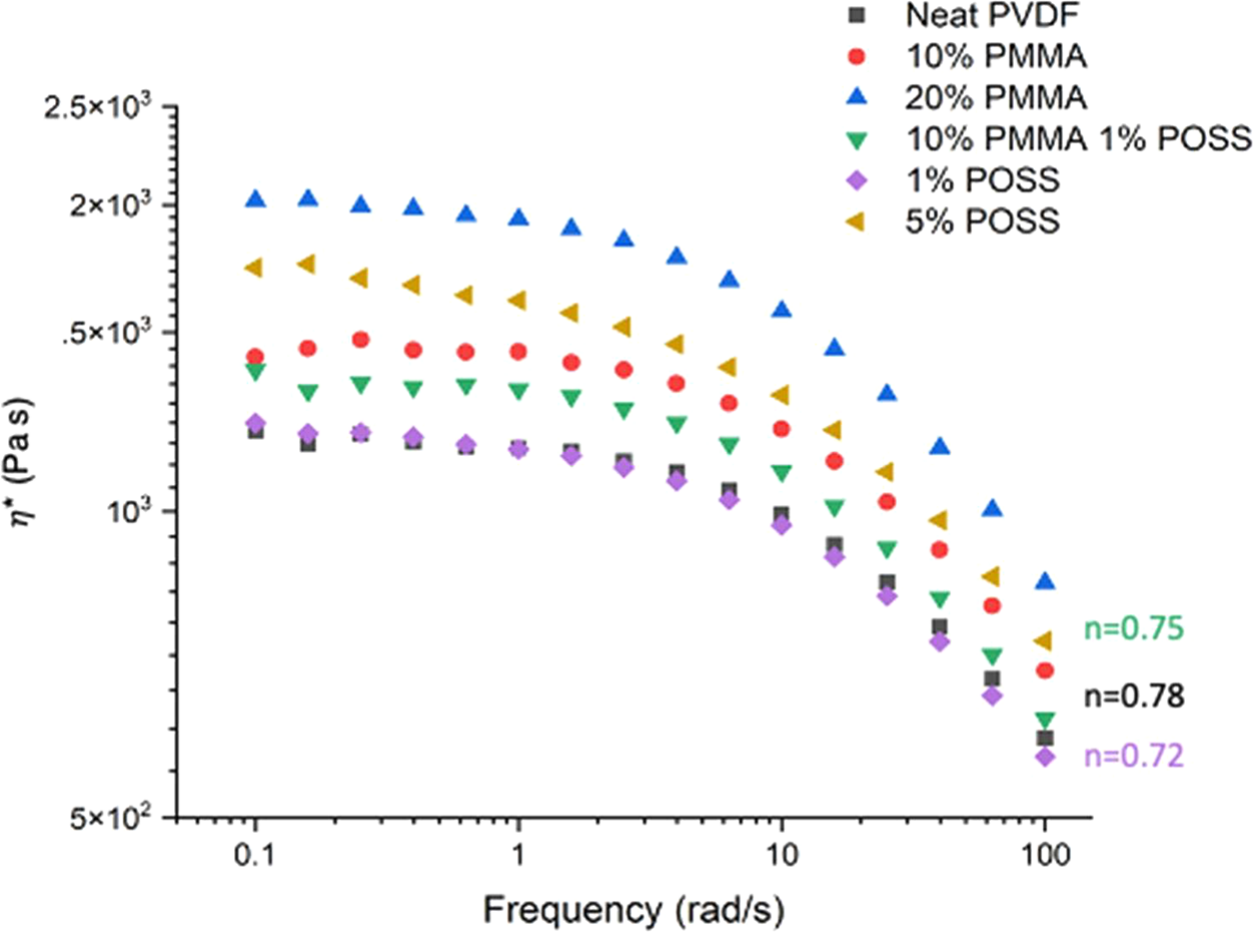
Complex viscosity as a function of shear rate for PVDF blends with PMMA and Cp-POSS at 220 °C. The power law index for the three down-selected blends for 3D printing are included.

Small amplitude oscillatory shear rheometry frequency sweeps were used to evaluate viscosity as a function of shear rate for the PVDF blends at the processing temperature, 220 °C (Figure 2). Zero shear viscosity of the 1 wt % Cp-POSS blend is similar that of neat PVDF, but all other blends show higher zero shear viscosity. The PVDF employed in this study is a high flow (low molecular weight) material, and the PMMA is of higher molecular weight, which explains the increase in zero shear viscosity. A power law index (n) was obtained by fitting the shear thinning region of the viscosity profile of each blend to a power law model (Table S4).^50^ All blends show increased shear thinning (smaller n), with 1 wt % Cp-POSS having the greatest effect. Shear thinning is critical because it impacts the consistency and the infill percentage of the MEX printed parts.

### 3.3 3D Printing

Based on crystallinity and shear thinning behavior, three samples were down selected for MEX printing studies: neat PVDF, 1 wt % Cp-POSS/PVDF, and 10 wt % PMMA/1 wt % Cp-POSS/PVDF. Neat PVDF has a total β-phase content of 37% and power law index of n = 0.78. The 1 wt % Cp-POSS/PVDF binary blend has a similar total β-phase content (36%) and improved shear thinning behavior (n = 0.72). The 10 wt % PMMA/1 wt % Cp-POSS/PVDF has a lower total β-phase content (27%), improved shear thinning (n = 0.75), and increased amorphous phase, which is expected to improve printability. The down-selected samples were fabricated into 3D printer filament using twin screw extrusion, and 3D printed test bars were used to evaluate warpage, interlayer adhesion, morphology, crystallinity, and piezoelectric performance.

#### 3.3.1 Sample Warpage

**Figure 3:**
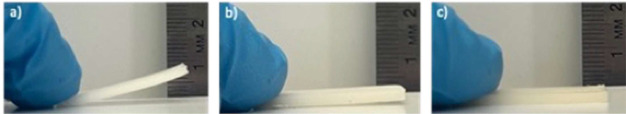
Warpage of 3D printed PVDF blends: (a) neat PVDF, (b) 1 wt % Cp-POSS/PVDF, and (c) 10 wt % PMMA/1 wt % Cp-POSS/PVDF.

Each of the filament samples were printed using the same conditions, including the use of a heated print bed to slow part cooling and increase crystallinity. Images of MEX printed parts are shown in Figure 3. For the neat PVDF, extensive warpage is observed, which is reduced significantly with the addition of Cp-POSS and PMMA. The improvements seen with only 1 wt % Cp-POSS are likely due to the reduction in melt viscosity under shear allowing more consistent deposition of each print layer and increasing the infill of the printed parts. Further improvements observed with 10 wt % PMMA are attributed to reduced shrinkage upon cooling due to lower overall crystallinity.

#### 3.3.2 Filament Morphology

**Figure 4:**
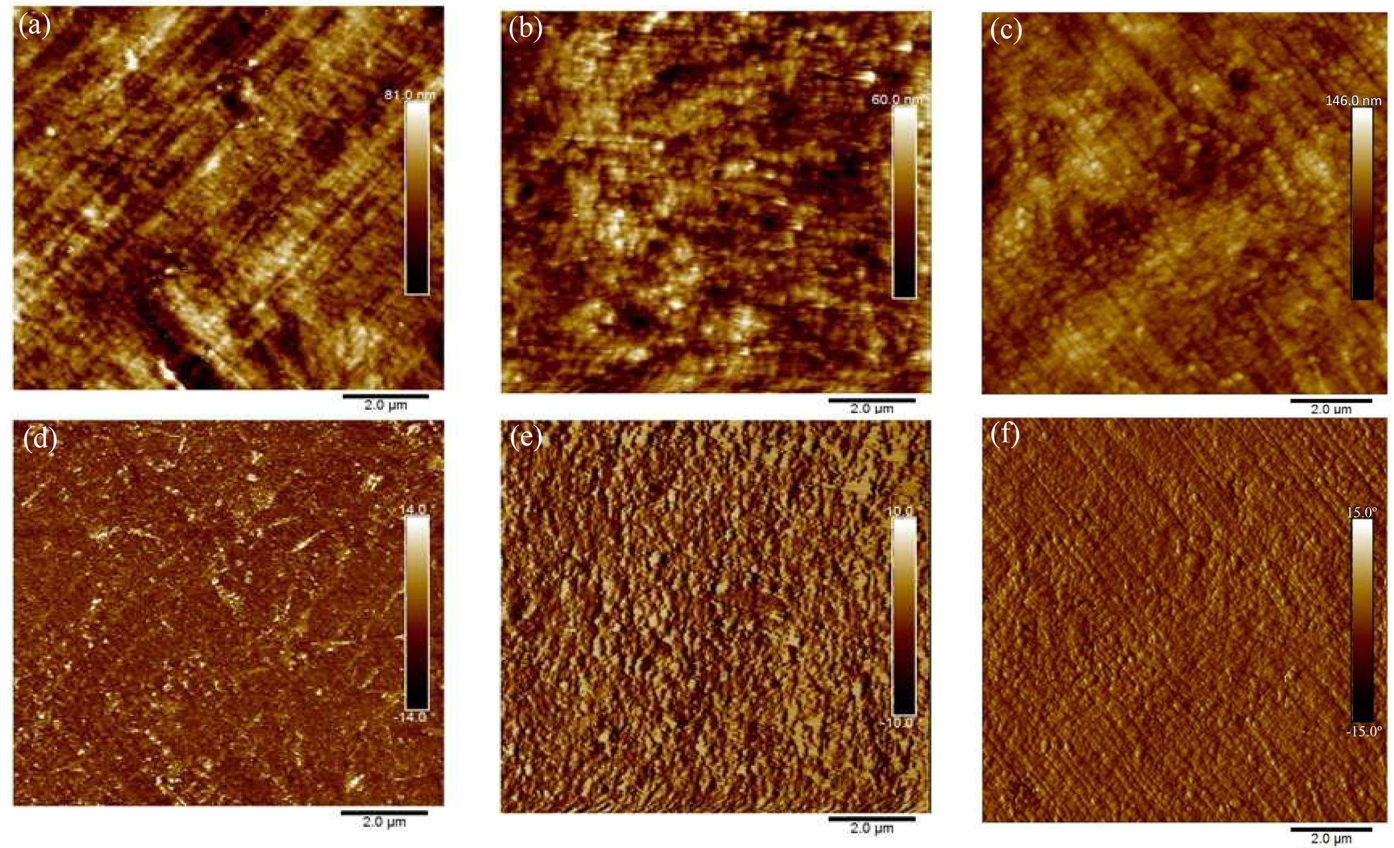
Atomic force microscopy of MEX printer filament cross sections. Height: (a) neat PVDF, (b) 1 wt % Cp-POSS/PVDF, and (c) 10 wt % PMMA/1 wt % Cp-POSS/PVDF. Phase: (d) Neat PVDF, (e) 1 wt % Cp-POSS/PVDF, and (f) 10 wt % PMMA/1 wt % Cp-POSS/PVDF. Cp-POSS aggregation, but no PMMA/PVDF phase separation, is observed.

Bulk morphologies were evaluated using AFM to image the cross sections of the printer filaments. Height images of the three filaments are similar, with no distinct phase separation and similar roughness (RMS values: neat PVDF-13 nm, 1 wt % Cp-POSS/PVDF-17 nm and 10 wt % PMMA/1 wt % Cp-POSS/PVDF-11 nm) (Figure 4a-c). Phase images also show no obvious phase separation, but a more textured surface is observed for the Cp-POSS-containing samples, likely related to dispersed Cp-POSS particles (Figure 4d-f).^35^ A high level of dispersion is also apparent in SEM-EDX images, where no Si element is observed for neat PVDF, but blends show areas rich in Si indicative of Cp-POSS (Figure S7). Lack of observable phase separation of PMMA and PVDF is consistent with previous reports of good miscibility of the polymers during melt compounding.^27^

#### 3.3.3 Interlayer Adhesion

**Figure 5:**
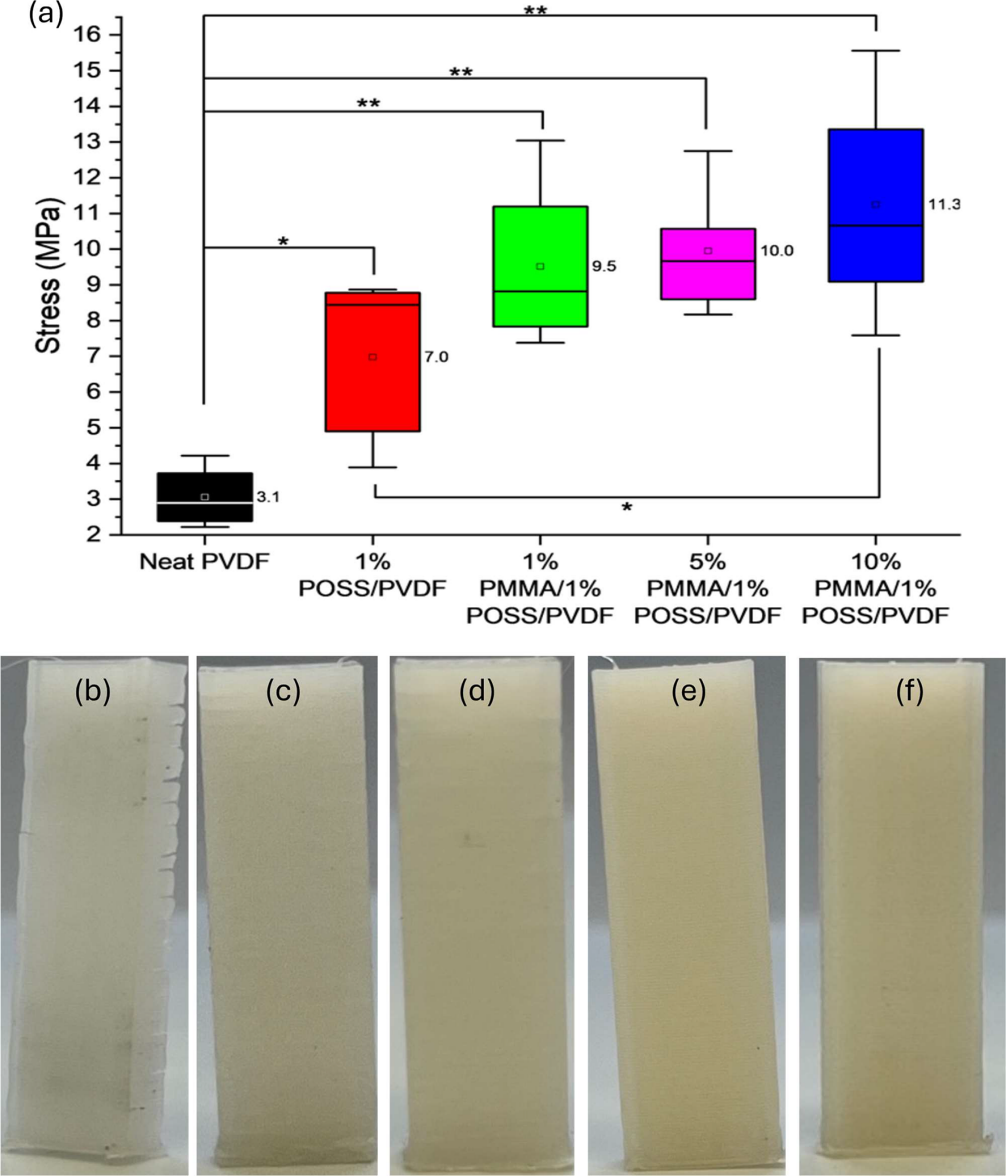
(a) Stress required to separate layers of MEX printed PVDF blends when measured in tension perpendicular to printing direction. Printed test samples of (b) neat PVDF, (c) 1 wt % Cp-POSS/PVDF, (d) 1 wt % PMMA/1 wt % Cp-POSS/PVDF, (e) 5 wt % PMMA/1 wt % Cp-POSS/PVDF, and (f) 10 wt % PMMA/1 wt % Cp-POSS/PVDF. Asterisks indicate sample averages are statistically different by *t* test (*p* < 0.05). Printed neat PVDF shows clear separation between layers.

Based on the improvements observed in 3D printing with the ternary blends, two additional ternary blends containing 1 and 5 wt % PMMA with 1 wt % Cp-POSS were prepared for mechanical and piezoelectric property evaluation. Testing of samples in tension perpendicular to the MEX layer direction was performed to determine the stress required to achieve adhesive failure between the layers. Significant increases are observed in average stress at interlayer adhesion failure for all blends in comparison to neat PVDF, doubling on incorporation of 1 wt % Cp-POSS and increasing almost 4-fold for the ternary blend with 10 wt % PMMA/1 wt % Cp-POSS (Figure 5a and Table S5). The 1 and 5 wt % PMMA ternary blends give no statistically significant improvement in stress at interlayer adhesion failure in comparison to the 1 wt % POSS binary blend. Printed neat PVDF parts show visible separation between the layers, caused by PVDF crystallinity and low surface energy (Figure 5b). No such layer separation is observed for the binary and ternary blends (Figure 5c-f). Improvements in the binary blend containing 1 wt % Cp-POSS are likely related to improved print infill and print accuracy with the improved flow of this blend, allowing greater interfacial interaction.^51^ The further improvements with the addition of PMMA are likely related to the increase in amorphous content, which reduced shrinkage and warpage during recrystallization.

#### 3.3.4 Crystallinity of Printed Parts

**Figure 6:**
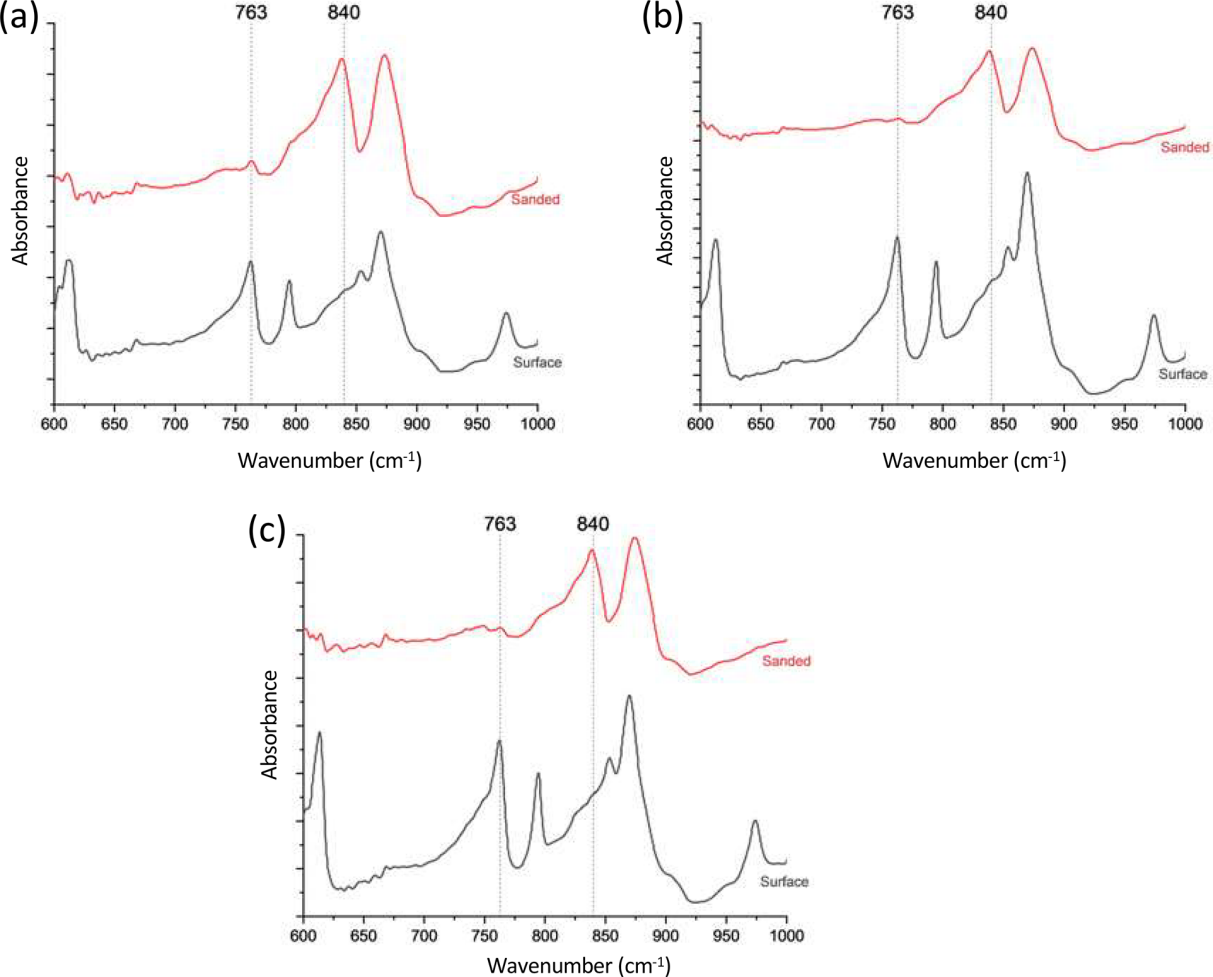
ATR-FTIR spectra of 3D printed samples before and after surface removal. (a) neat PVDF (b) 1 wt % Cp-POSS/PVDF (c) 10 wt % PMMA/1 wt % Cp-POSS/PVDF. β allomorph is observed in all sanded samples.

ATR-FTIR and DSC were used to evaluate the degree of crystallinity, F_β-phase,_ and total β-phase content of the 3D printed parts. Figure 6a-c shows the ATR-FTIR spectra of the 3D printed parts before and after the removal of the surface layer. An all-α phase surface layer (peak at 763 cm^–1^) is formed during the printing process as expected, and upon removal of the surface, the peak associated with the β allomorph is apparent (840 cm^–1^).

Table 2 is used to compare the measured *X*_c_, F_β-phase_, and total β-phase content of MEX printed samples. Degree of crystallinity values are consistent with melt pressed samples and are dependent on the amount of PMMA in the blends. F_β-phase_ and total β-phase content are higher for the neat PVDF, 1 wt % Cp-POSS, and 1 wt % PMMA/1 wt % Cp-POSS samples compared to the samples containing higher levels of PMMA. F_β-phase_ is slightly higher for the printed parts compared to the molded parts, which may be related to shear induced alignment of the polymer chains during the printing process. Total β-phase content decreases with increased incorporation of PMMA in the blends, as is expected due to the increase in amorphous content.

**Table 2:** Percent Crystallinity, Percent β Allomorph, and Total β Phase Content of MEX Printed PVDF Blends

Sample	*X*_c_	F_β-phase_	Total β-phase content
FFF-Neat PVDF	55%	71%	38%
FFF-% POSS/PVDF	57%	70%	40%
FFF-1% PMMA/1% POSS/PVDF	54%	71%	38%
FFF-5% PMMA/1% POSS/PVDF	55%	69%	36%
FFF-10% PMMA/1% POSS/PVDF	52%	66%	34%

#### 3.3.5 Piezoelectric Performance

**Figure 7:**
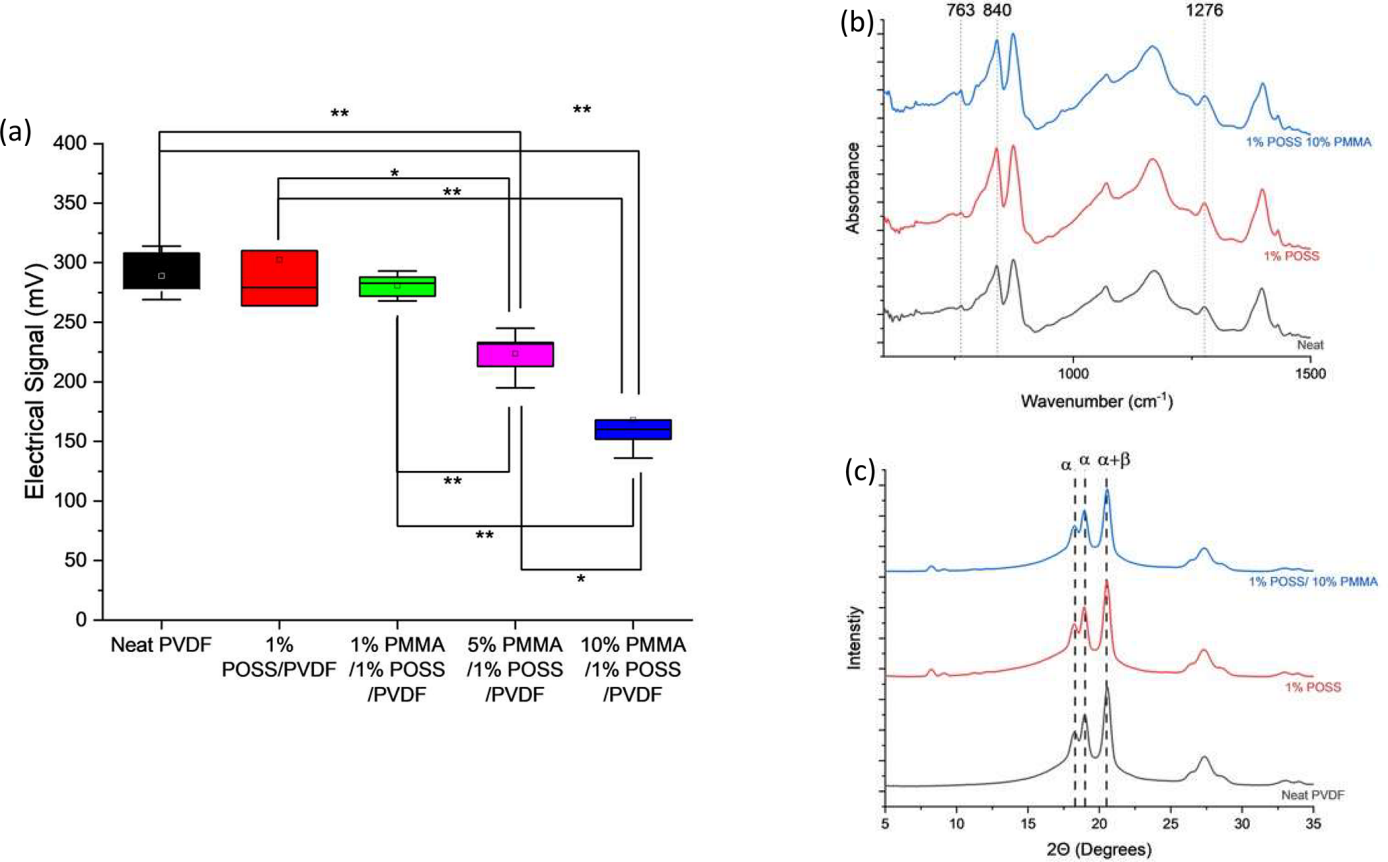
(a) Piezoelectric performance, (b) ATR-FTIR spectra, and (c) WAXS spectra of MEX printed PVDF blends. Asterisks indicate samples are statistically different by *t* test (*p* < 0.05). 5% and 10% PMMA samples show reduced piezoelectric response related to lower β allomorph concentration. β allomorph is observed in all printed samples.

The β-phase, with its all-trans conformation and high dipole moment, is responsible for the piezoelectric response of PVDF.^18^ Piezoelectric performance of the 3D printed parts was measured by applying a controlled mechanical force and measuring the electrical response. Figure 7a shows average piezoelectric response of the printed parts, and the results are consistent with the β-phase content of the blends. PVDF, 1 wt % Cp-POSS, and 1 wt % PMMA/1 wt % Cp-POSS samples have similar total β-phase content and show no statistically significant difference in piezoelectric response. Increases in PMMA concentration to 5 and 10 wt % yield significant reductions in piezoelectric performance, in-line with decreases in total β-phase content in these ternary blends. Piezoelectric coefficients, d_33_, are tabulated in Table S6, and reveal the same trends. An unsanded neat PVDF sample is included in the table for reference, and its average is within experimental error of the measured response for the sanded neat PVDF. Figure 7b shows ATR-FTIR spectra of the printed parts after the removal of the surface layer, where the peaks consistent with the β-phase allomorph are observed in all samples. The WAXS scattering patterns for the printed parts show three major peaks, two related to the α polymorph (18.3°, 19.0°) and one related to the mix of the α and β allomorphs (20.5°). Differentiation between α and β allomorphs using WAXS is difficult in mixed systems because the scattering peaks for the two allomorphs overlap.^17,43,47^ (Figure 7c) Both ATR-FTIR and WAXS indicate the presence of the β polymorph in all printed samples.

## 5 Conclusions

Binary and ternary blends of PVDF with Cp-POSS and PMMA were prepared via twin screw extrusion. Crystallinity, β-phase content, rheological properties, and MEX printability of the filament were evaluated, and printed part warpage, interfacial adhesion, and piezoelectric properties were determined. Surprisingly, incorporation of just 1% Cp-POSS yielded significant improvements in interfacial adhesion, with increase in stress at break from 3.1 MPa for neat PVDF to 7 MPa for the 1% Cp-POSS blend. Addition of 1% PMMA with 1% Cp-POSS produced further increase in interfacial adhesion to 9.5 MPa. A similar pattern was observed in warpage reduction, with higher levels of PMMA showing greater reductions in warpage. Printability improvements are attributed to the higher flow in the Cp-POSS/PVDF blends, which yields increased infill and print accuracy, allowing greater interfacial interactions. Addition of the amorphous PMMA results in reduced shrinkage and warpage in the PVDF blend. These dramatic improvements in MEX printability are achieved without diminishing PVDF piezoelectric performance. Directly printed 1% Cp-POSS/1% PMMA/PVDF blends yielded average piezoelectric coefficient of 24 pC/N, with no statistically significant difference in comparison to that of neat PVDF. Piezoelectric coefficient was reduced at higher PMMA loading levels; however, all blends exhibited a piezoelectric response and ATR-FTIR evaluation demonstrated greater than 50% β-phase content in the crystalline phase. These findings demonstrate that PVDF blends with piezoelectric response can be directly printed via MEX methods without the need for additional postprinting processing. This additive approach allowing improvements in printability while retaining piezoelectric performance is a simple platform that can be used to fabricate a wide range of piezoelectric materials for high value applications.

## Supporting Information Available

Figure S1: Structure of cyclopentyl polyhedral oligomeric silsesquioxane (Cp-POSS). Figure S2: Schematic of preparation process. Figure S3. Schematic of sample preparation and test setup for piezoelectric testing. Figure S4: ATR-FTIR spectra of sanded PVDF/Cp-POSS/PMMA blends 1% PMMA/PVDF, 5% PMMA/PVDF, 10% PMMA/PVDF, 20% PMMA/PVDF, 1% Cp-POSS/PVDF, 5% Cp-POSS/PVDF, 1% Cp-POSS/10% PMMA/PVDF, 5% Cp-POSS/10% PMMA/PVDF, and 5% Cp-POSS/20% PMMA/PVDF. Figure S5: F_β-phase_ as a function of surface layer removal for a neat PVDF sample produced via compression molding. Figure S6: Differential scanning calorimetry thermograms for the second heat of each of the PVDF blends. Figure S7: Scanning electron microscopy images show the surfaces neat PVDF, 1% Cp-POSS/PVDF, and 1% Cp-POSS/10% PMMA/PVDF samples. Table S1: Compounding parameters for blend compounding and filament fabrication. Table S2: MEX printing parameters used for the fabrication of piezoelectric parts and interlayer adhesion testing. Table S3: Crystallinity and β-phase content of PVDF blends crystallized via quenching and slow cooling. Table S4: Zero shear viscosity and calculated power law index values for PVDF/PMMA/Cp-POSS blends from small amplitude oscillatory shear rheology. Table S5: Average stress at interlayer adhesion failure for MEX printed samples of PVDF with Cp-POSS and PMMA. Table S6: Average piezoelectric coefficient (d_33_) calculated using the change generated from impact force during testing of MEX printed parts. (PDF)
