# Correction to “β-Phase Crystallinity, Printability, and Piezoelectric Characteristics of Polyvinylidene Fluoride (PVDF)/Poly(methyl methacrylate) (PMMA)/Cyclopentyl-polyhedral Oligomeric Silsesquioxane (Cp-POSS) Melt-Compounded Blends”

**DOI:** 10.1021/acsapm.4c01719

**Published:** 2024-06-20

**Authors:** Toby R. Edwards, Rahul Shankar, Paul G. H. Smith, Jacob A. Cross, Zoe A. B. Lequeux, Lisa K. Kemp, Zhe Qiang, Scott T. Iacono, Sarah E. Morgan

In the original article, the eighth author’s name was misspelled as Scott T. Iacano. It should read Scott T. Iacono.

