# Supplementary material for: β-Phase Crystallinity, Printability, and Piezoelectric Characteristics of Polyvinylidene Fluoride (PVDF)/Poly(methyl methacrylate) (PMMA)/Cyclopentyl-Polyhedral Oligomeric Silsesquioxane (Cp-POSS) Melt-Compounded Blends
Source: ACS Appl Polym Mater. 2024 May 14;6(10):5803–13. doi: 10.1021/acsapm.4c00468 (PMC11129178; doi:10.1021/acsapm.4c00468)
Supplement: Supplementary file 1 — ap4c00468_si_001.pdf [file ap4c00468_si_001.pdf]

Supporting Information

β-phase crystallinity, printability, and piezoelectric characteristics of polyvinylidene fluoride (PVDF)/polymethyl methacrylate (PMMA)/cyclopentyl-polyhedral oligomeric silsesquioxane (Cp-POSS) melt-compounded blends.

Toby R. Edwards[1], Rahul Shankar[1], Paul G. H. Smith[1], Jacob A. Cross[1], Zoe A. B. Lequeux[1], Lisa K. Kemp[1], Zhe Qiang[1], Scott T. Iacano[2], Sarah E. Morgan[1,]*

1 School of Polymer Science and Engineering, University of Southern Mississippi, 118 College Drive, #5050, Hattiesburg, Mississippi, 39406, United States

2 Department of Chemistry and Chemistry Research Center, United States Air Force Academy, 2355 Fairchild Drive, Suite 2N225, Colorado Springs, Colorado, 80840, United States

* Sarah.morgan@usm.edu; 601-266-5296

Supplementary Materials:

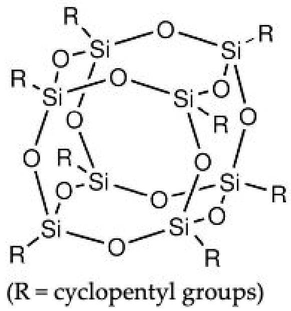

Figure S1. Structure of cyclopentyl polyhedral oligomeric silsesquioxane (Cp-POSS).

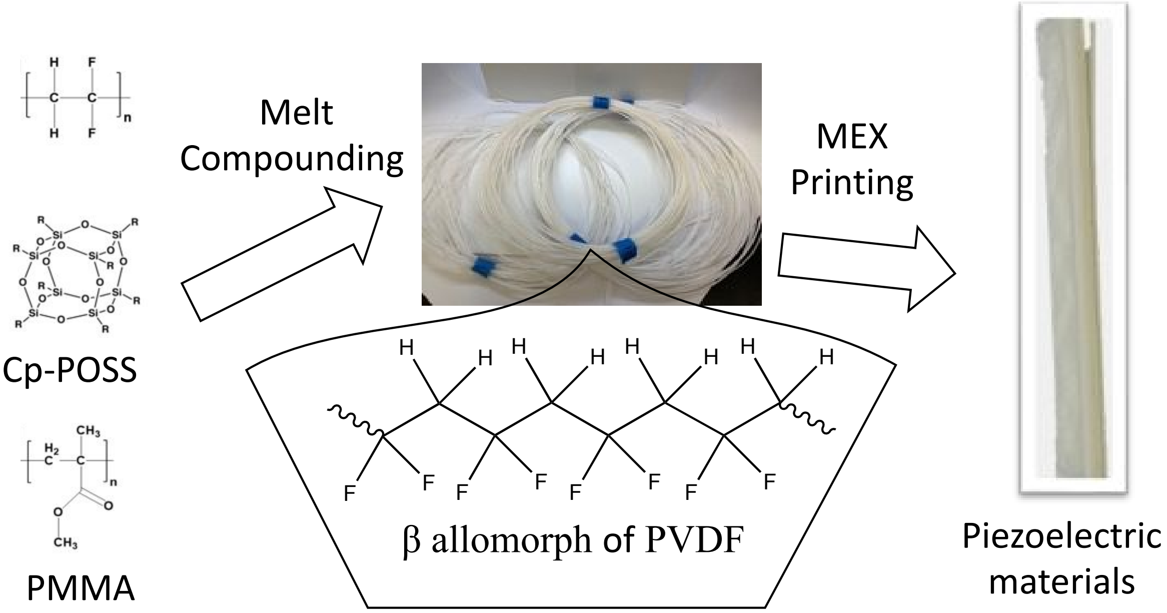

Figure S2. Schematic of preparation process.

Table S1. Compounding parameters for blend compounding and filament fabrication.

Parameter	Setting
Screw Speed	100 RPM
L/D	40
Temperature Profile	190-230 °C
Volumetric Feed Speed	12 RPM
Pellet Size	2 mm
Strand Thickness	2.5 ± 0.36 mm

Table S2. MEX printing parameters used for the fabrication of piezoelectric parts and interlayer adhesion testing.

Parameter	Setting
Nozzle diameter	0.5 mm
Nozzle temperature	230 °C
Print bed temperature	80 °C
Layer height	0.2 mm
Print speed	20 mm/s
Fan speed	10 %
Walls	2
Top/bottom layers	4
Infill pattern	Grid
Raster angle	45°, 135°
Infill density	80 %
Bed adhesion	Brim

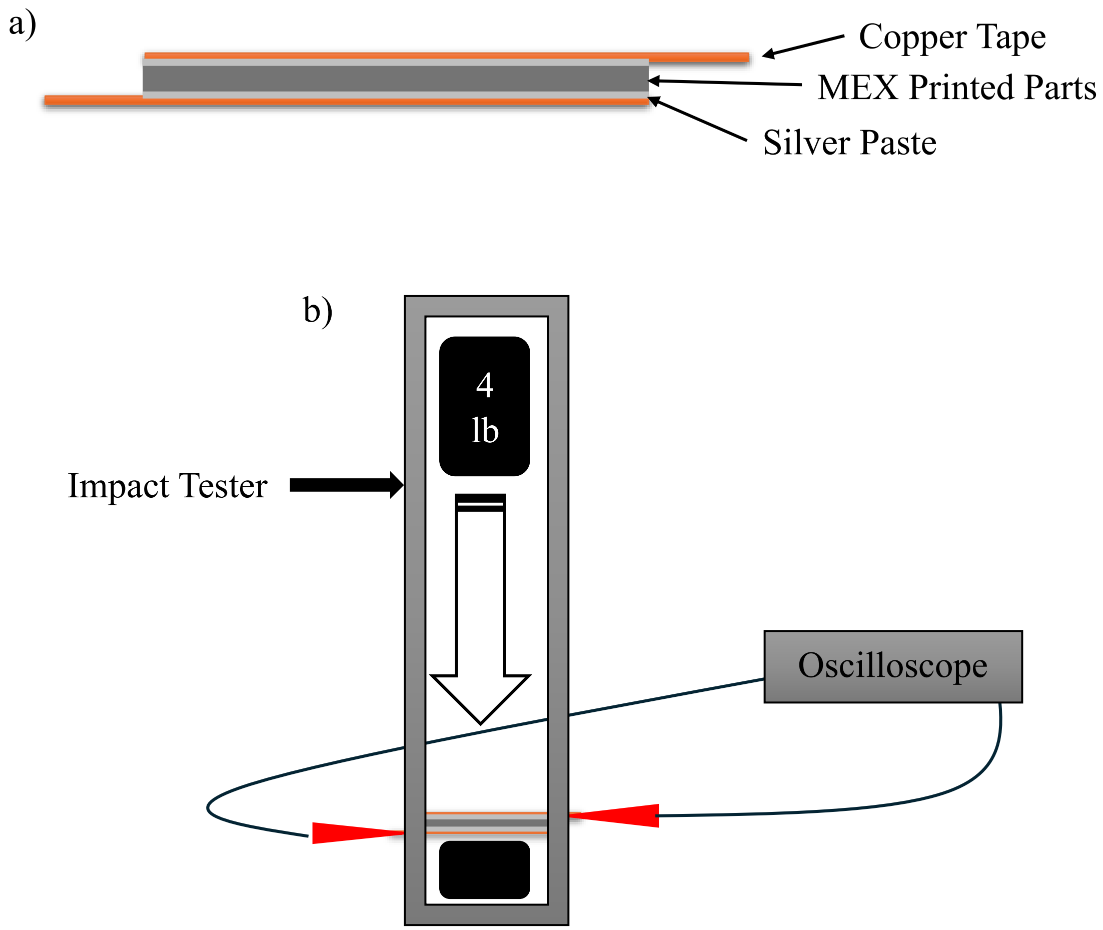

Figure S3. Schematic of sample preparation for piezoelectric testing. (a) Sample preparation for piezoelectric testing, the surface of the MEX printed parts were sanded and silver paste was applied as conductive layer, copper tape was then applied on top of the silver paste. The samples were insulated with electrical tape prior to testing. (b) The MEX printed samples were placed into an impact tester and alligator clips were attached to the copper electrodes. Voltage responses from impact force were measured using and oscilloscope. The 4 lb weight was dropped from a defined height and the maximum voltage output was recorded for each sample.

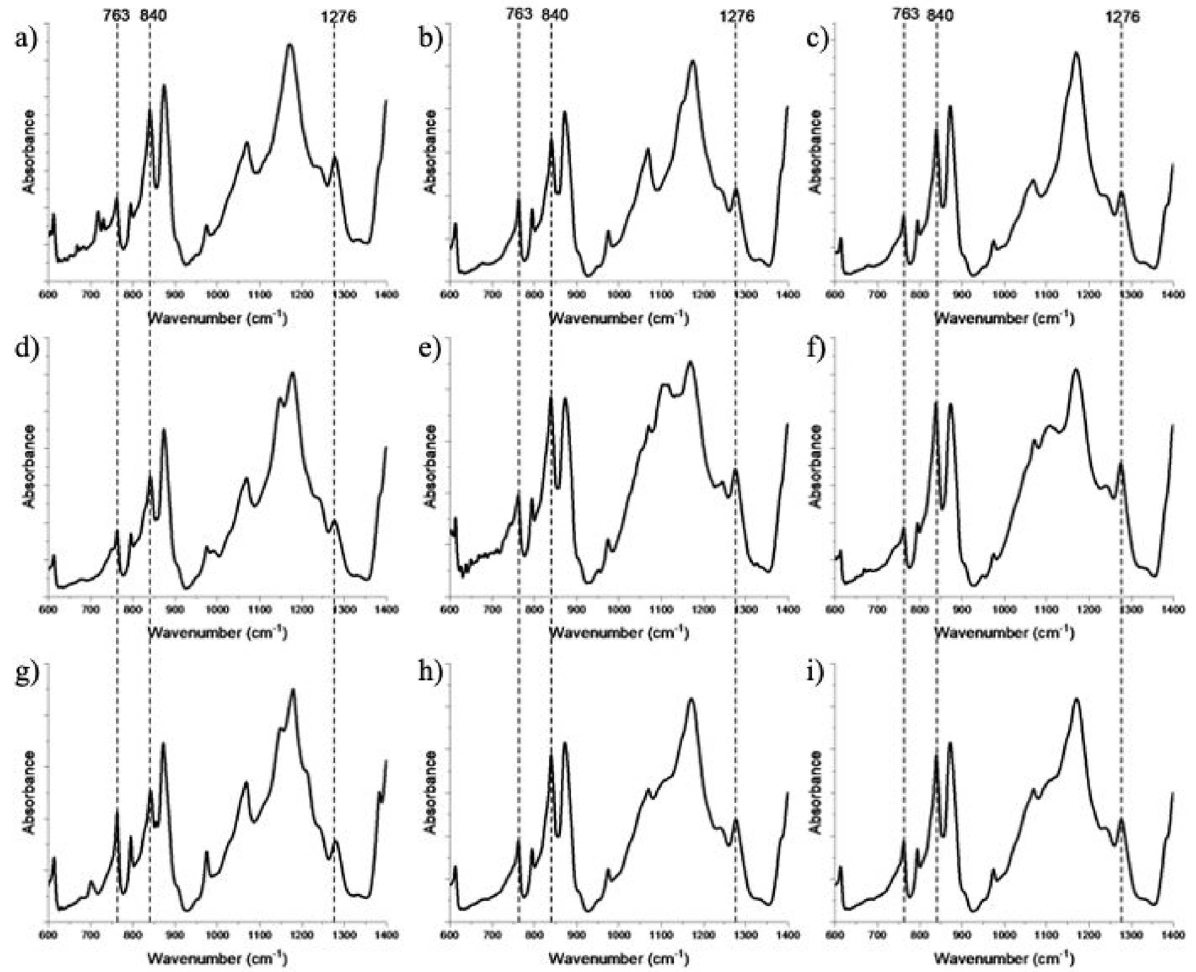

Figure S4. FTIR-ATR spectra of sanded PVDF/Cp-POSS/PMMA blends (a) 1 wt.% PMMA/PVDF (b) 5 wt.% PMMA/PVDF (c) 10 wt.% PMMA/PVDF (d) 20 wt.% PMMA/PVDF (e) 1 wt.% Cp-POSS/PVDF (f) 5 wt.% Cp-POSS/PVDF (g) 1 wt.% Cp-POSS/10 wt.% PMMA/PVDF (h) 5 wt.% Cp-POSS/10 wt.% PMMA/PVDF (i) 5 wt.% Cp-POSS/20 wt.% PMMA/PVDF.

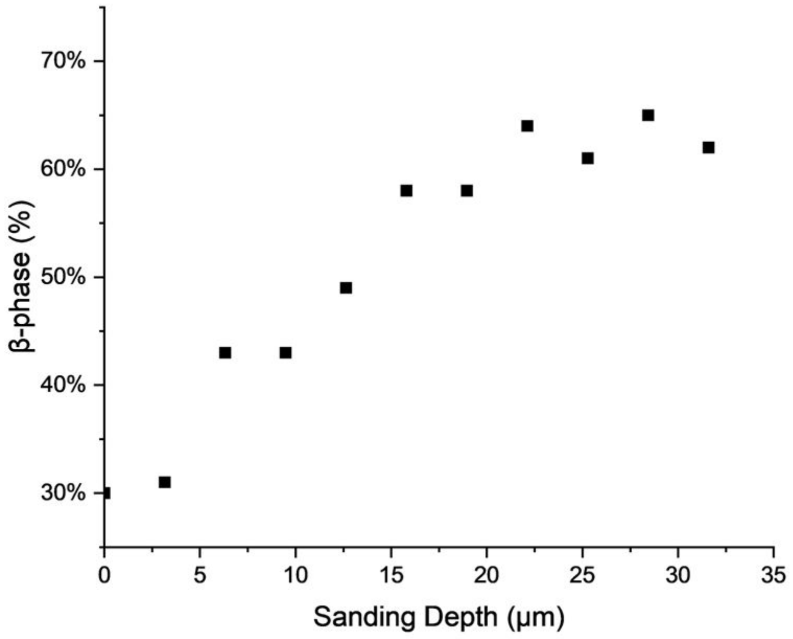

Figure S5. $F_{\beta\text{-phase}}$ as a function of surface layer removal for a neat PVDF sample produced via compression molding. The β- phase percentage is calculated using the absorbances at 763 cm^{-1} and 840 cm^{-1}; the intensity of the peak at 840 cm^{-1} increases with sanding depth.

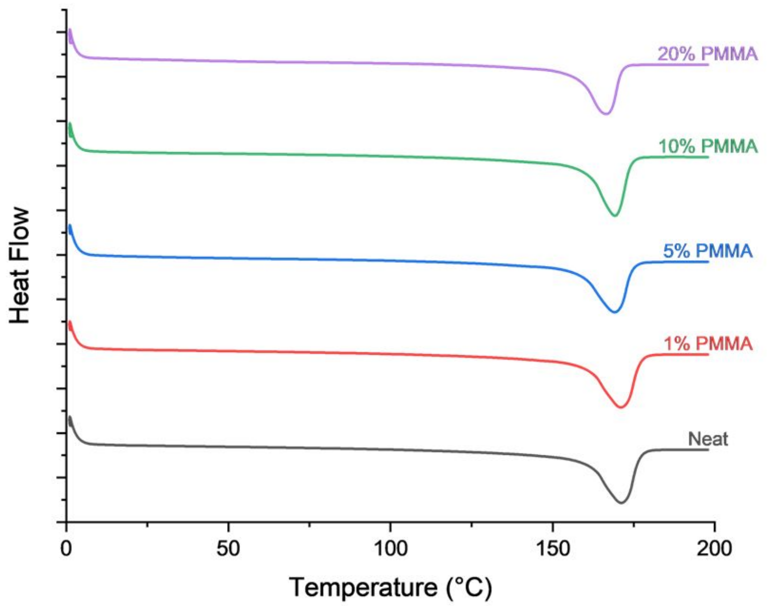

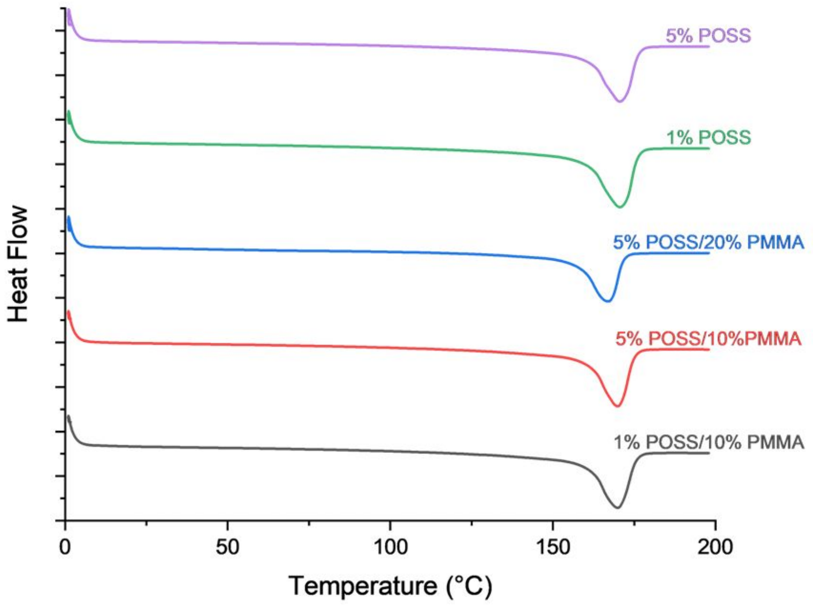

Figure S6. Differential scanning calorimetry thermograms for the second heat of each of the PVDF blends.

Table S3. Crystallinity and beta phase content of PVDF blends crystallized via quenching and slow cooling.

**Sample**	**X_c**	**$F_{\beta\text{-phase}}$**	**Total β-phase Content**
Neat PVDF Quenched	52%	63%	32%
Neat PVDF Slow Cooled	56%	65%	36%
1% POSS/PVDF Quenched	49%	68%	33%
1% POSS/PVDF Slow Cooled	59%	69%	41%
10% PMMA/1% POSS/PVDF Quenched	44%	65%	29%
10% PMMA/ 1% POSS/PVDF Slow Cooled	52%	63%	33%

Table S4. Zero shear viscosity and calculated power law index values for PVDF/PMMA/POSS blends from small amplitude oscillatory shear rheology.

Sample	Power law Index (n)	Zero Shear Viscosity (Pa-s)
Neat PVDF	0.78	1150
1% PMMA/PVDF	0.77	1130
5% PMMA/PVDF	0.76	1560
10wt% PMMA/PVDF	0.76	1940
20wt% PMMA/PVDF	0.73	1320
1% POSS/PVDF	0.72	1150
5% POSS/PVDF	0.76	1610
1% POSS/10% PMMA/PVDF	0.75	1320
5% POSS/10% PMMA/PVDF	0.75	1700
5% POSS/20% PMMA/PVDF	0.75	2130

Table S5. Average stress at interlayer adhesion failure for MEX printed samples of PVDF with Cp-POSS and PMMA.

Sample	Average Stress at Adhesion Failure (MPA)	Standard Deviation
MEX- Neat PVDF	3.1	0.9
MEX- 1% POSS/PVDF	7.0	2.4
MEX- 1% PMMA/1% POSS/PVDF	9.5	2.5
MEX- 5% PMMA/1% POSS/PVDF	10.0	1.8
MEX- 10% PMMA/1% POSS/PVDF	11.3	3.2

Table S6. Average piezoelectric coefficient (d_{33}) calculated using the change generated from impact force during testing of MEX printed parts, also included in a neat PVDF sample that was not sanded prior to testing.

Sample	d_{33}(pC/N)	Standard Deviation
MEX-Unsanded Neat PVDF	23.4	2.9
MEX- Neat PVDF	25.5	1.8
MEX- 1% POSS/PVDF	26.7	4.9
MEX- 1% PMMA/1% POSS/PVDF	24.0	2.0
MEX- 5% PMMA/1% POSS/PVDF	18.6	2.4
MEX- 10% PMMA/1% POSS/PVDF	23.4	2.9

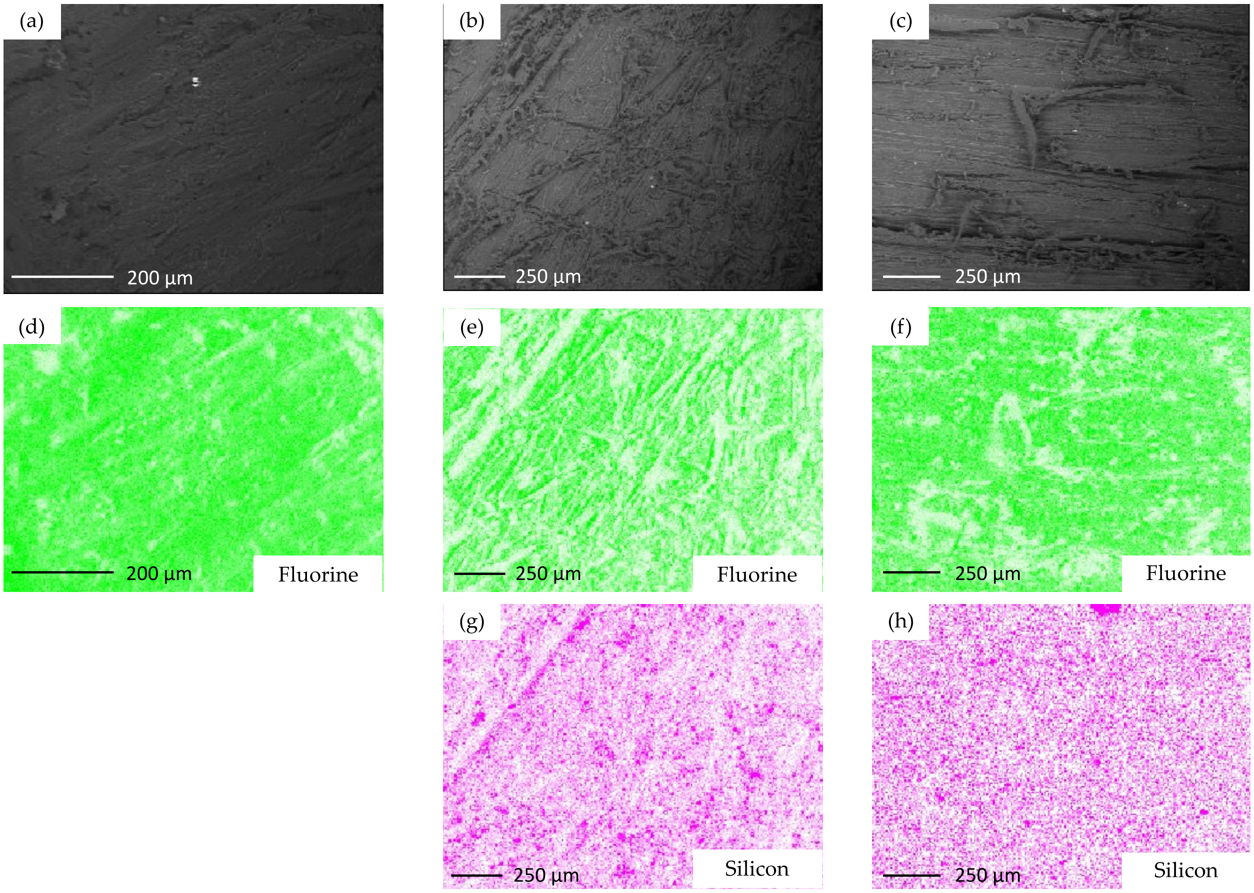

Figure S7. Scanning electron microscopy images show the surfaces of: (a) neat PVDF (b) 1 wt.% Cp-POSS/PVDF (c) 1 wt.% Cp-POSS/10 wt.% PMMA/ PVDF samples. Elemental mapping of the fluorine shows PVDF present throughout (d) neat PVDF (e) 1 wt.% Cp-POSS/PVDF (f) 1 wt.% Cp-POSS/10% PMMA/ PVDF. No silicon was detected in the neat PVDF sample (a). Si is observed throughout the(g) 1 wt.% Cp-POSS/PVDF and (h) 1 wt.% Cp-POSS/10% PMMA/PVDF samples representing dispersed POSS particles.
